# XuanBi Mitigates Rheumatoid Arthritis via Regulation of ECM‐Receptor Interactions and IL‐17 Signaling: Insights From Integrated Proteomic and Pharmacological Analyses

**DOI:** 10.1002/fsn3.72498

**Published:** 2026-10-25

**Authors:** Hao Zhu, Zhenfeng Pan, Ying Lu, Ping Xia, Weiming He, Jingqi Zeng, Fan Wang, Xiao Liu, Minggang Wei

**Affiliations:** ^1^ Department of Traditional Chinese Medicine The First Affiliated Hospital of Soochow University, Soochow University Suzhou China; ^2^ Department of Pharmacy The First Affiliated Hospital of Soochow University, Soochow University Suzhou China; ^3^ Department of Integrated Traditional Chinese and Western Medicine Dushu Lake Hospital, Soochow University Suzhou China; ^4^ Department of Nephrology Jiangsu Province Hospital of Chinese Medicine Nanjing China; ^5^ Department of Orthopedics The Second Affiliated Hospital of Hunan University of Chinese Medicine (Hunan Traditional Chinese Medical Hospital) Changsha China; ^6^ Department of Burn and Plastic Surgery The First Affiliated Hospital of Soochow University, Soochow University Suzhou China

**Keywords:** bioinformatics analysis, collagen induced arthritis, network pharmacology, synovial hyperplasia, synovial inflammation, XuanBi

## Abstract

Plant‐derived polyphenols possess broad anti‐inflammatory activities and may benefit chronic inflammatory diseases. XuanBi is a traditional Chinese medicine formula containing orally absorbable polyphenolic constituents and is used to treat rheumatoid arthritis (RA). This study investigated its absorbable components and potential mechanisms associated with synovial inflammatory hyperplasia. XuanBi was evaluated in collagen‐induced arthritis (CIA) rats and rheumatoid arthritis synovial fibroblasts (RASFs). Constituents in the decoction and rat serum were characterized by high‐performance liquid chromatography coupled with quadrupole‐Orbitrap mass spectrometry (HPLC‐Q‐Orbitrap‐MS). Predicted targets were intersected with synovial differentially expressed proteins (DEPs), followed by network pharmacology, Gene Ontology (GO), Kyoto Encyclopedia of Genes and Genomes (KEGG) enrichment analyses, and experimental validation. Twenty‐six absorbable constituents were identified, including catechin, quercetin, baicalein, and wogonin, together with 14 candidate proteins associated with RA synovial pathology. Enrichment analysis implicated extracellular matrix (ECM)–receptor interaction, focal adhesion, and interleukin‐17 (IL‐17) signaling. Medium‐ and high‐dose XuanBi reduced joint swelling, synovial hyperplasia, and inflammatory infiltration in CIA rats. In RASFs, XuanBi inhibited proliferation, migration, and invasion. It also downregulated fibronectin 1 (FN1) and caveolin 1 (CAV1), reduced phosphorylation of SRC, focal adhesion kinase (FAK), and FYN, decreased integrin alpha 2b (ITGA2B)^+^/integrin beta 1 (ITGB1)^+^ cells, and reduced interleukin‐1 beta (IL‐1β), interleukin‐6 (IL‐6), tumor necrosis factor alpha (TNF‐α), matrix metallopeptidase 3 (MMP3), and matrix metallopeptidase 9 (MMP9). Overall, XuanBi exerted anti‐inflammatory and anti‐synovial‐hyperplasia effects in experimental RA, accompanied by changes in proteins associated with these pathways, supporting the potential involvement of these pathways and warranting further mechanistic and clinical investigation.

## Graphical abstract

The anti‐inflammatory effect of XuanBi Herbal Formula was evaluated in arthritic rat models, in which suppression of synovial inflammation was observed. The chemical ingredients of XuanBi were detected in both the decoction and rat peripheral blood. Potential targets of the formula were then intersected with previous synovial proteomic data, through which core targets and associated signaling pathways were predicted. Subsequently, the effects of XuanBi Herbal Formula on rheumatoid arthritis synovial fibroblasts were further investigated in vitro. Validation of the core targets and pathway‐related proteins further suggested that the inhibitory effects of XuanBi on synovial inflammatory proliferation may be associated with modulation of the ECM‐receptor interaction, focal adhesion, and IL‐17 signaling pathway.

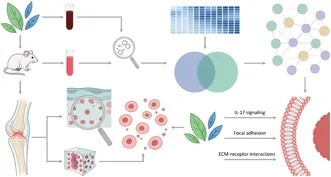

## 1 Introduction

Inflammatory hyperplasia of the synovial tissue has been identified as an important pathological feature of rheumatoid arthritis (RA) (Lerner et al. 2018). This process triggers the production of various pro‐inflammatory mediators (Guo et al. 2024), which in turn exacerbate systemic inflammation and symptoms of pain and swelling in the joints (Menzel et al. 2022). Differential protein expression is a major pathogenic event in the pathogenesis of RA, and the regulatory processes controlling protein expression in the synovium are not well understood (Kumar et al. 2023). Elucidating the regulatory processes is important in identifying new disease processes (Su et al. 2024), genes (Bonaventura et al. 2018), and targets (Mihaylova et al. 2024). In our previous study, we employed the application of isobaric tags for relative and absolute quantification (iTRAQ) to investigate the proteomic profile of the synovial tissues of healthy rats and those with collagen‐induced arthritis (CIA) (Ma and Zhou 2024). This study identified 154 differentially expressed proteins (DEPs) that were associated with RA pathology. Therefore, based on the previous findings, we investigated potential molecular mechanisms associated with the effects of traditional Chinese medicine in experimental RA.

In China, traditional Chinese medicine (TCM) has been known for its potential to relieve the clinical manifestations of RA for centuries (Knizkova et al. 2022). TCM is characterized by its holistic approach to treating diseases, and it presents an alternative therapeutic approach with the classical XuanBi formula being of clinical significance (Jia et al. 2023). Previous studies have reported potential therapeutic effects of XuanBi in RA (Xu 2024). XuanBi, which dates back to the classical medical book titled Warm Diseases published in 1798 (Alturaiki et al. 2022), has also been used clinically to alleviate RA‐related symptoms, including joint redness, swelling, heat, and pain (Miyashita et al. 2022). The XuanBi formula consists of Stephania tetrandra root (15 g), *Prunus armeniaca* seed (15 g), *Forsythia suspensa* fruit (9 g), *Gardenia jasminoides* fruit (9 g), *Coix lacryma‐jobi* seed (15 g), *Pinellia ternata* root (9 g), *Bombyx mori* excrementum (9 g), *Vigna angularis* seed (9 g), and Talcum (15 g) (Figure 1A). Plant identification was confirmed by reference to the latest revision of the online publication of the World Flora Online. Given the botanical composition of XuanBi, plant‐derived polyphenolic compounds may represent an important group of its bioactive constituents. Polyphenols have attracted considerable attention because of their antioxidant and anti‐inflammatory properties and their potential roles in chronic inflammatory diseases. Representative polyphenols identified in medicinal and dietary plants have also shown RA‐related biological activities (Javadi et al. 2017; You et al. 2024).

**FIGURE 1:**
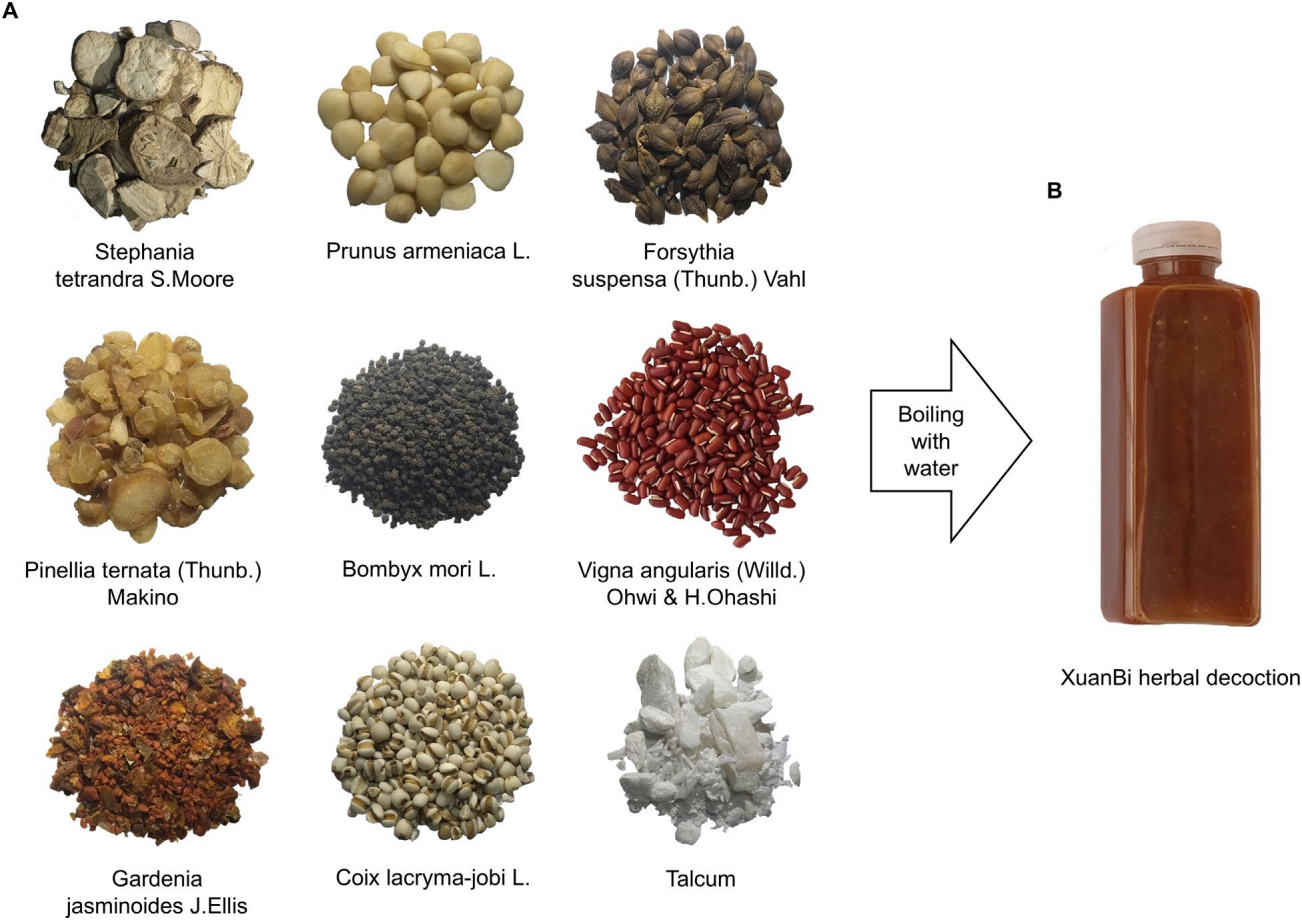
Preparation of XuanBi herbal decoction. (A) XuanBi herbal formula, including 7 herbs (Stephania tetrandra root, *Prunus armeniaca* seed, *Forsythia suspensa* fruit, *Pinellia ternata* root, *Vigna angularis* seed, *Gardenia jasminoides* fruit, *Coix lacryma‐jobi* seed.), 1 mineral ingredient (Talcum), and 1 animal excrement ( *Bombyx mori* excrementum). (B) XuanBi herbal decoction.

Modern scientific studies demonstrated that XuanBi reduces serum levels of major inflammatory mediators, including tumor necrosis factor alpha (TNF‐α), interleukin‐6 (IL‐6), and interleukin‐17 (IL‐17), in RA model animals, and reduces levels of matrix metalloproteinases (Wen et al. 2024). Although the therapeutic potential of XuanBi has been demonstrated in preliminary studies, scientific research that explores the underlying mechanisms of its effectiveness in RA treatment remains scarce (Malemud 2017). Notably absent in this line of research are studies that use synovial tissue and relevant cell types in the context of RA. XuanBi is a multi‐component traditional Chinese medicinal formula with potential pleiotropic effects on multiple pathological processes in RA (Bian et al. 2023). Network pharmacology prediction suggested that XuanBi may exert anti‐arthritic effects, at least in part, by modulating signaling pathways such as the IL‐17 pathway (Bian et al. 2023). However, the predictions need experimental verification in appropriate animal and cell models. A more comprehensive understanding of the pharmacological activities of XuanBi is therefore needed. Such an understanding may improve the characterization of the pharmacological properties and therapeutic potential of XuanBi and provide a basis for its further preclinical and clinical evaluation in RA. Further studies are required to determine whether XuanBi could eventually complement conventional pharmacological therapies. By scientifically proving traditional remedies using modern scientific methods, it will be possible to bring classical and modern medicine together (Zhou et al. 2024), which will aid in the evidence‐based use of effective ancient therapeutic modalities for rheumatologic diseases (Zhang 2020).

Following previous proteomic studies (Ma and Zhou 2024), the present investigation aimed to integrate bioinformatics and network pharmacology to identify candidate proteins and explore potential molecular pathways associated with the effects of XuanBi. The investigation centers on the basic pathological process of inflammatory synovial hyperplasia in RA. This research investigated the potential molecular actions of XuanBi and explored how its therapeutic effects may be associated with synovial inflammatory hyperplasia‐related pathways, thereby providing experimental evidence for its further preclinical evaluation in RA.

## 2 Materials and Methods

### 2.1 Arthritic Model Replication

Thirty‐six Sprague–Dawley rats aged 6–7 weeks, including 18 males and 18 females, with a mean body weight of 200 ± 30 g, were purchased from the Laboratory Animal Center of Soochow University. Six rats were randomly assigned to the normal control group, and the remaining 30 rats were used to establish the collagen‐induced arthritis (CIA) model. After successful model establishment, the CIA rats were randomly divided into five groups, with six rats in each group: CIA model group, low‐dose XuanBi group (LD), medium‐dose XuanBi group (MD), high‐dose XuanBi group (HD), and methylprednisolone group (MP). Equal sex distribution was maintained as far as possible across groups. Sex‐stratified analyses were not performed because the study was not designed or powered to evaluate sex‐specific treatment effects. Following Chondrex Inc.'s guidelines, we established the CIA model. For initial induction, we mixed bovine Type II collagen (2 mg/mL, Chondrex, US) with complete Freund's adjuvant (10 mL, Sigma–Aldrich, US) to prepare mixture A. Researchers formulated mixture B for the booster by blending an equal volume of bovine Type II collagen with 10 mL of incomplete Freund's adjuvant (Sigma–Aldrich, USA). On the first day, researchers injected 0.2 mL of mixture A subcutaneously at the tail base of rats assigned to the model group. Subsequently, on the seventh day, the same group received a 0.1 mL injection of mixture B via the same procedure. On the 28th day, both normal and CIA rats were euthanized using CO_2_ asphyxiation with the gradual filling method at a flow rate of 10%–30% of the chamber volume per minute, ensuring minimal distress. Subsequently, ankle joints and synovial tissues from knee joints were aseptically harvested. The research team carried out all animal experiments following national regulations for laboratory animal care, and the procedures were approved by the Ethics Committee of Soochow University (license No. SUDA20220613A01).

### 2.2 Experimental Design for XuanBi Intervention in CIA Rats

Following successful collagen‐induced arthritis (CIA) model establishment, rats were randomly assigned to the following groups, with six rats in each group: normal control group, CIA model group, low‐dose XuanBi group (LD), medium‐dose XuanBi group (MD), high‐dose XuanBi group (HD), and methylprednisolone group (MP). Rats in the normal control and CIA model groups received no therapeutic intervention except for the corresponding vehicle administration. XuanBi intervention was initiated after the booster immunization. The clinical adult dosage of the XuanBi herbal formula was 105 g/day, calculated based on an adult body weight of 70 kg. After conversion to the equivalent rat dose, the medium dose was set at 9.3 g/kg/day. The low and high doses were set at half and twice the medium dose, corresponding to 4.65 and 18.6 g/kg/day, respectively. XuanBi decoction was administered by oral gavage once daily for 3 weeks. Rats in the MP group received methylprednisolone at 2 mg/kg by intraperitoneal injection every other day and were used as the positive control group. At the end of the treatment period, rats were euthanized using the aforementioned protocol. Serum samples and ankle joint tissues were collected for subsequent high‐performance liquid chromatography coupled with quadrupole‐Orbitrap mass spectrometry (HPLC‐Q‐Orbitrap‐MS), histopathological analysis, and immunohistochemical assessment.

### 2.3 Morphology

The hind limb conditions of the rats were assessed and monitored for their body weight in grams each week. The arthritis index and the maximum joint sizes in millimeters of the rats were also noted. Photographs were taken to observe any changes in joint morphology, and joint redness and swelling were evaluated for the arthritis index using a rating scale: 0—absence of swelling and redness; 1—minor swelling or redness limited to individual digits; 2—moderate swelling in ankle, moderate redness; 3—severe swelling of whole paw, severe redness; 4—maximum swelling of multiple joints, maximum redness. Each paw was scored independently, and the arthritis index for each rat was calculated as the sum of the scores for all four paws, yielding a total score ranging from 0 to 16, with higher scores indicating greater arthritis severity.

### 2.4 Histopathology

Researchers fixed their ankle joints in 4% paraformaldehyde at 4°C for 24 h. They treated their samples with 10% ethylenediaminetetraacetic acid (EDTA) for decalcification for 21 days, followed by dehydration using a series of alcohols, clearing with xylene, and finally embedding in paraffin. Sections of 5 μm were cut and mounted. Histopathological changes were evaluated by hematoxylin and eosin (H&E) staining, whereas proteoglycan and glycosaminoglycan content were evaluated by safranin O and toluidine blue staining, respectively. A Leica DFC425C microscope was used to capture images.

### 2.5 HPLC‐Q‐Orbitrap‐MS Analysis

The composition of XuanBi herbal decoction and its serum metabolites were identified by HPLC‐Q‐Orbitrap‐MS. The sample preparation of 200 μL of herbal decoction and 50 μL of serum was performed by protein precipitation with methanol (4:1 v/v), followed by vortexing for 10 min, ultrasonic extraction for 5 min, and centrifugation at 13,000 rpm for 10 min. Chromatographic separation was achieved using an AQ‐C18 column (150 × 2.1 mm, 1.8 μm) operated at 35°C, eluting for 30 min using a 0.1% formic acid in water (A) and methanol (B) linear gradient elution at 0.3 mL/min. Mass spectrometry was carried out using a Q Exactive Orbitrap spectrometer with an electrospray ionization (ESI) source in positive and negative ionization modes (spray voltage 3.2 kV, capillary temperature 300°C). Full‐scan MS data was acquired at a resolution of 70,000 (m/z 100–1500), and data‐dependent MS^2^ spectra were collected at a resolution of 17,500 with stepped normalized collision energy (NCE) values of 30, 40, and 60.

### 2.6 Bioinformatics Analyses

The Traditional Chinese Medicine Systems Pharmacology database and analysis platform (TCMSP; https://tcmsp‐e.com/tcmsp.php) was used to retrieve candidate compounds and their predicted targets for each herb in the XuanBi formula. TCMSP provides pharmacokinetic and systems pharmacology information, including oral bioavailability (OB), drug‐likeness (DL), compound‐related targets, and disease associations, and has been widely used for network pharmacology‐based studies of traditional Chinese medicine formulas. Compounds were preliminarily screened using OB > 30% and DL > 0.18, which are commonly used thresholds in TCMSP‐based analyses for identifying compounds with relatively favorable oral exposure and drug‐like properties (Wang et al. 2024; Zheng et al. 2023). To improve the biological relevance of the predicted component‐target network, we further focused on components detected by HPLC‐Q‐Orbitrap‐MS in both XuanBi decoction and serum samples after oral administration. Predicted targets corresponding to these absorbable components were collected from TCMSP and used to construct the herb‐component‐target network in Cytoscape 3.10. These predicted component‐related targets were then intersected with previously identified differentially expressed proteins (DEPs) in collagen‐induced arthritis (CIA) synovial tissue. The overlapping proteins were regarded as candidate proteins potentially associated with the effects of XuanBi on CIA‐related synovial pathological changes. We carried out Gene Ontology (GO) and Kyoto Encyclopedia of Genes and Genomes (KEGG) enrichment analyses using ClueGo (version 2.5.1) implemented in Cytoscape.

### 2.7 Immunohistochemistry (IHC)

Ankle joints were fixed in 4% paraformaldehyde for 24 h, decalcified in 10% EDTA for 21 days, and embedded in paraffin. Sections (5 μm) were subjected to heat‐induced epitope retrieval in 10 mM citrate buffer (pH 6.0) for 15 min. After blocking with 5% normal goat serum in phosphate‐buffered saline (PBS), sections were incubated overnight at 4°C with primary antibodies against matrix metallopeptidase 9 (MMP9), matrix metallopeptidase 3 (MMP3), fibronectin 1 (FN1), and caveolin 1 (CAV1) (Servicebio, China). After washing, biotinylated horseradish peroxidase (HRP)‐conjugated secondary antibodies were applied, and signals were developed using 3,3′‐diaminobenzidine (DAB), followed by hematoxylin counterstaining. Sections were subsequently dehydrated, cleared, and mounted.

### 2.8 Isolation, Culturing, and Identification of Rat Synovial Fibroblasts

Synovial tissues were carefully harvested from knee joints for further study. Synovial fibroblasts (SF) of normal rats and rheumatoid arthritis synovial fibroblasts (RASF) of CIA rats were isolated and cultured by using collagenase digestion. The cells were then fixed in 4% paraformaldehyde. Subsequently, the cells were permeabilized using 0.5% Triton X‐100. To prevent nonspecific binding, 10% goat serum was used. The cells were then incubated with the vimentin antibody, 1:1000, overnight. The next day, the secondary antibody, 1:200, was used. After the second overnight incubation, the nuclei of the cells were stained using 4′,6‐diamidino‐2‐phenylindole (DAPI). Immunofluorescence staining was done to identify the fibroblasts (Chen et al. 2014; Cubuk et al. 2024).

### 2.9 Experimental Design for XuanBi Intervention in RASFs

After morphological observation and vimentin immunofluorescence identification, synovial fibroblasts isolated from normal rats (SFs) and rheumatoid arthritis synovial fibroblasts isolated from CIA rats (RASFs) were used for the in vitro experiments. Cells at passage 3 were used to ensure experimental consistency. SFs isolated from normal rats served as the control group (Ctrl), whereas RASFs isolated from CIA rats were used to establish the model and treatment groups. For evaluation of extracellular matrix (ECM)–receptor interaction and focal adhesion signaling, RASFs in the Model group received no additional stimulation. The treatment groups were exposed to XuanBi herbal decoction at the predetermined working concentrations corresponding to the medium‐dose (MD) and high‐dose (HD) groups. Arg‐Gly‐Asp‐Ser (RGDS, 75 μM) and Daidzein (25 μM) were used as FN1 inhibitor and CAV1 inhibitor, respectively.

For evaluation of the IL‐17 signaling pathway, an IL‐17‐activated inflammatory environment was established in vitro because isolated RASFs lack the complete inflammatory microenvironment present in arthritic joints. RASFs in the Model, MD, and HD groups were first stimulated with recombinant rat interleukin‐17A (IL‐17A, 50 ng/mL, Servicebio, China) for 24 h, whereas SFs in the Ctrl group were not exposed to IL‐17A. Following the 24‐h IL‐17A stimulation, RASFs in the MD and HD groups were subsequently treated with the corresponding medium or high concentration of XuanBi for 7 days. RASFs in the Model group received no XuanBi treatment during the corresponding 7‐day period. The culture medium containing the corresponding XuanBi concentration was renewed every other day. This experimental design was used to evaluate the effect of XuanBi on RASFs following IL‐17A‐induced inflammatory activation. All in vitro experiments were performed using cells derived from at least three independent isolations. For 5‐Ethynyl‐2′‐deoxyuridine (EdU) staining, Transwell assays, wound‐healing assays, flow cytometry, western blotting, and ELISA, “n” represented independent biological replicates rather than repeated measurements from the same well, unless otherwise specified. For western blotting and ELISA, protein lysates and culture supernatants were collected from independently treated RASF cultures.

### 2.10 EdU Staining

We treated the cells with 10 μM EdU for 2 h at 37°C. After fixation with 4% paraformaldehyde, the cells were permeabilized using 0.3% Triton X‐100. EdU incorporation was then detected using a click chemistry kit. The cells were washed, stained with DAPI, and mounted. Fluorescence microscopy was used to capture images of the cells, which were then used to quantify the number of EdU‐positive cells to measure proliferation.

### 2.11 Transwell Migration and Invasion Assay

We dissociated cells using 0.25% trypsin and plated them in the upper chamber of a Transwell insert at a density of 3.0 × 10^5^ cells/mL. The upper compartment of the wells was treated with serum‐free media (control) or media containing XuanBi or inhibitors, while the lower compartment contained complete media. After 24 h, the cells were fixed with 4% paraformaldehyde, stained with 0.1% crystal violet, and then visualized. ImageJ software was used for image analysis.

We prepared the upper chamber by coating it with 60 μL of diluted Matrigel and allowing it to solidify. After introducing 100 μL of serum‐free medium, cells were plated at 3.0 × 10^5^ cells/mL. The upper chamber was then supplied with serum‐free medium (control) or medium containing XuanBi and the respective inhibitors. The lower chamber was supplied with complete medium, and the culture was allowed to proceed for 48 h. After the culture, the cells were fixed, stained, and photographed, followed by analysis with ImageJ software.

### 2.12 Wound Healing Assay

Cells were dissociated using 0.25% trypsin–EDTA and plated at a cell density of 5.0 × 10^5^ cells/mL (1 mL/well) into six‐well plates. The plates were gently shaken and left to incubate for 24 h. The control and model groups were treated with serum‐reduced medium, while the treatment groups were treated with serum‐reduced medium plus XuanBi or inhibitors. Wound images were taken to calculate area using ImageJ software.

### 2.13 Flow Cytometry (FCM)

After treatment, cells from each group were harvested for flow cytometric analysis. Cells were washed twice with phosphate‐buffered saline (PBS), resuspended as single‐cell suspensions, and incubated with antibodies against integrin alpha 2b (ITGA2B) and integrin beta 1 (ITGB1) according to the manufacturer's instructions. After staining, cells were washed and analyzed using flow cytometry. The proportions of ITGA2B^+^/ITGB1^+^ and ITGA2B^−^/ITGB1^−^ cells were quantified to assess the effects of XuanBi treatment on integrin expression in RASFs. Each experiment was performed using at least three independent biological replicates.

### 2.14 Western Blot (WB)

After treatment, total protein was extracted from independently treated SFs or RASFs using radioimmunoprecipitation assay (RIPA) buffer supplemented with protease and phosphatase inhibitors. Protein concentrations were determined using a bicinchoninic acid (BCA) assay. Equal amounts of protein (20–30 μg) were separated by sodium dodecyl sulfate–polyacrylamide gel electrophoresis (SDS‐PAGE) and transferred onto polyvinylidene fluoride (PVDF) membranes. After blocking with 5% non‐fat milk for 1 h at room temperature, membranes were incubated overnight at 4°C with primary antibodies against FN1 and CAV1 (Servicebio, China), p‐FYN, p‐SRC, p‐FAK, and p‐P38 (Thermo Fisher Scientific, USA), p‐Act1 (Invitrogen, USA), and p‐NF‐κB p65 (Sigma‐Aldrich, USA). After washing, membranes were incubated with HRP‐conjugated secondary antibodies for 1 h at room temperature. Protein bands were visualized using enhanced chemiluminescence (ECL) and quantified with ImageJ. WB analyses were performed using at least three independent biological replicates.

### 2.15 Enzyme‐Linked Immunosorbent Assay (ELISA)

After treatment, culture supernatants from independently treated RASF cultures were collected and centrifuged to remove cell debris. The concentrations of MMP3 (Abcam, UK), MMP9, IL‐1β, IL‐6, and TNF‐α (R&D Systems, USA) were measured using commercial ELISA kits according to the manufacturers' instructions. Briefly, samples and standards were added to the assay plates and processed according to the respective manufacturers' protocols. Absorbance was measured using a microplate reader, and analyte concentrations were calculated from the corresponding standard curves. ELISA assays were performed using at least three independent biological replicates.

### 2.16 Statistical Analyses

Statistical analyses were performed using SPSS version 27.0, and graphs were generated using GraphPad Prism version 9.5. Data are presented as the mean ± standard deviation (SD). Normality and homogeneity of variance were assessed using the Shapiro–Wilk test and Levene's test, respectively. For comparisons among multiple independent groups, one‐way analysis of variance (ANOVA) followed by Dunnett's post hoc test was performed, with each treatment group compared with the Model group. For arthritis index scores measured repeatedly over time, a two‐way repeated‐measures ANOVA was used to evaluate the effects of group, time, and their interaction, followed by multiple comparisons as appropriate. Statistical significance is represented as *, ** for *p* values < 0.05 and 0.01, respectively.

## 3 Results

### 3.1 XuanBi Inhibits Redness and Swelling in CIA Rats

We observed significant changes in the ankle joints of rats from each intervention group on Day 28 (Figure 2A). The control limb was slender with normal pink skin. In contrast, the arthritic model limb displayed clear signs of swelling, ankylosis, and hyperemia. Compared with model rats, CIA rats treated with MD or HD of XuanBi presented progressively improved symptoms, including reduced skin redness and decreased ankle swelling. However, the LD XuanBi treatment results were not significantly different. MP, which was used as a positive control, significantly reduced the skin redness and ankle swelling. These results are markedly different from those seen in the model group.

**FIGURE 2:**
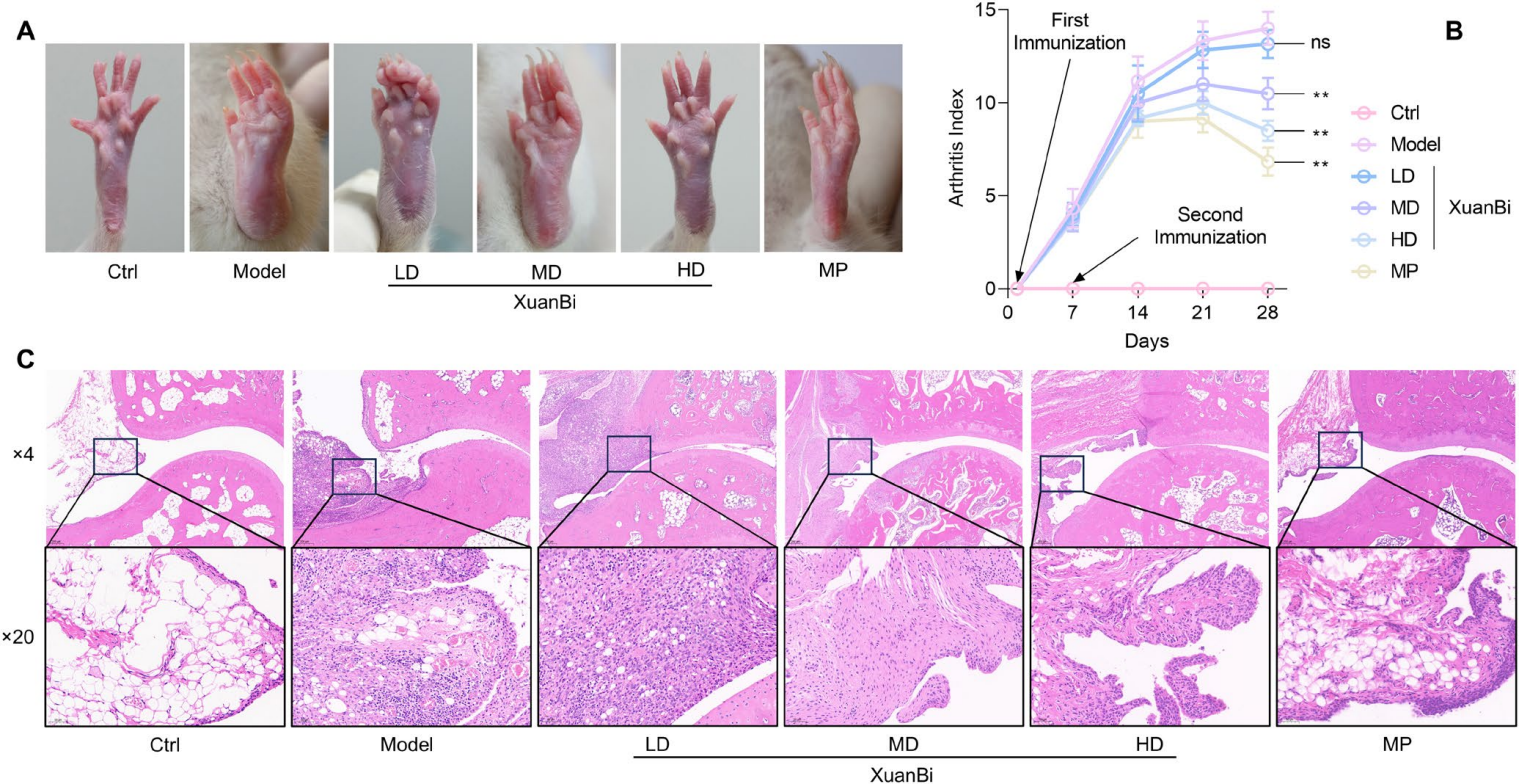
Comparative observation of the effects of XuanBi herbal formula on different intervention groups: Control (Ctrl), arthritic model (Model), methylprednisolone intervention (MP), and XuanBi intervention at low dose (LD), medium dose (MD), or high dose (HD). (A) Representative morphological change of the posterior limb in each group on Day 28. (B) Arthritis index changes in each group (*n* = 6) measured at seven‐day intervals. (C) Representative histopathological changes of ankle joint on Day 28. Statistical analysis was performed using two‐way repeated‐measures analysis of variance (ANOVA) followed by multiple comparisons as appropriate. ns, not significant; *p < 0.05; **p < 0.01.

As for the changes in arthritis index scores (Figure 2B), all groups showed a trend of consistent increase from Day 7 to Day 21 after immunization except for the control group. From Day 21 to Day 28, arthritis index scores in the model and LD XuanBi groups steadily increased without significant differences between them. On the other hand, the MD and HD XuanBi groups experienced a significant reduction in arthritis index, which was notably different from the model group (*p* < 0.01). MP, as the positive control, exhibited the most pronounced decrease in arthritis index among all treatment groups (*p* < 0.01).

### 3.2 XuanBi Inhibits Inflammatory Hyperplasia in the Synovium of CIA Rats

At Day 28, we observed varying pathological changes in the ankle joints of each group (Figure 2C). The normal ankle joint exhibited a distinct gap within the joint and appeared normal with minimal inflammatory cell infiltration. The arthritic model exhibited pronounced synovial hyperplasia and a significant presence of inflammatory cell infiltration. The LD XuanBi group showed similar histopathological features as the model group. Treatment with MD and HD XuanBi, however, effectively mitigated synovial proliferation and inflammatory infiltration. The use of MP as a positive control had the most pronounced effect on reducing these conditions.

### 3.3 Identification of Absorbable Components of the XuanBi Herbal Formula

HPLC‐Q‐Orbitrap‐MS analysis of the XuanBi formula decoction and rat serum samples enabled the identification with high mass accuracy (< 3 ppm), following comparison and screening against the TCMSP database, a total of 26 ingredients were confirmed as absorbable components (Figure 3, Table 1): 5‐Hydroxymethyl‐2‐furaldehyde, (‐)‐Catechin, (‐)‐Stepholidine, quercetin, luteolin, baicalein, wogonin, coniferin, liquiritin, licochalcone B, onjixanthone I, 2‐Monoolein, β‐Amyrin acetate, hesperetin, ammidin, glabridin, cycloartenol, (‐)‐Phillygenin, mandenol, crocetin, tetraneurin a, linoleic acid, beta‐sitosterol, oleic acid, stigmasterol, geniposidic acid. Each ingredient's characterization was achieved through retention time alignment, exact mass determination, and MS/MS fragmentation patterns.

**FIGURE 3:**
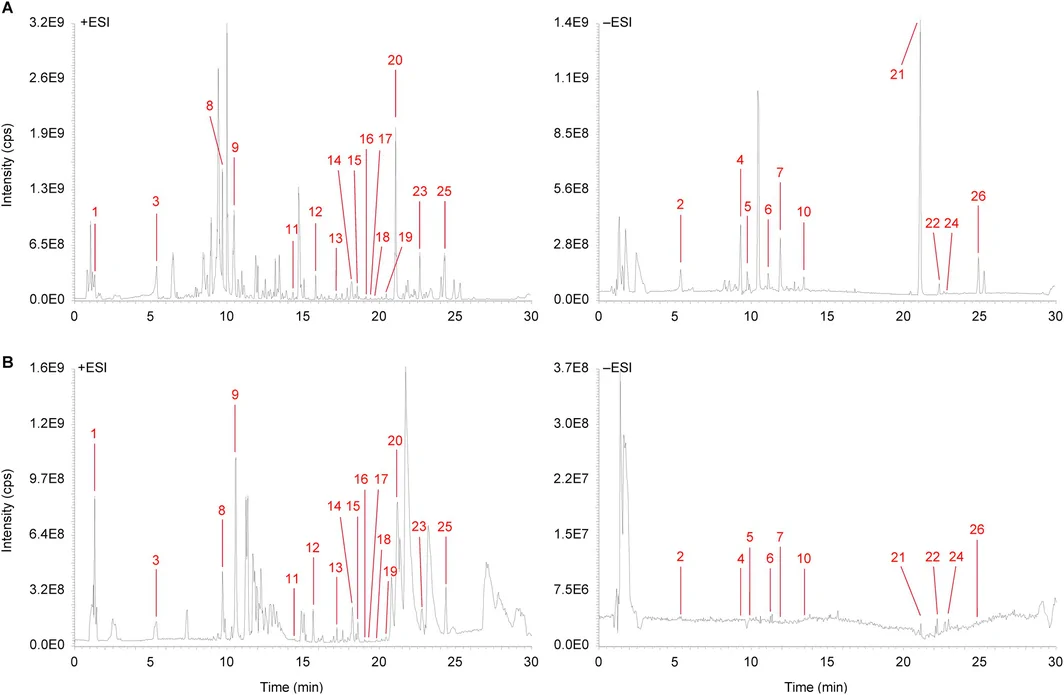
Total ion chromatogram acquired in both positive and negative electrospray ionization (ESI) mode. (A) XuanBi herbal decoction. (B) Rat serum after oral administration of XuanBi herbal decoction.

**TABLE 1:** The 26 absorbable components identified in both XuanBi herbal decoction and rat serum following oral administration of the decoction.

No.	RT (min)	Name	Formula	Reference Ion	m/z	ppm	Source
1	1.36	5‐Hydroxymethyl‐2‐furaldehyde	C_6_H_6_O_3_	[M + H] +	125.02	1.2	*Pinellia ternata* root
2	5.20	(‐)‐Catechin	C_15_H_14_O_6_	[M − H] −	289.07	−0.64	*Vigna angularis* seed
3	5.47	(‐)‐Stepholidine	C_19_H_19_NO_4_	[M + H] +	326.13	2.0	*Prunus armeniaca* seed
4	6.80	Quercetin	C_15_H_10_O_7_	[M − H] −	301.03	−0.8	*Forsythia suspensa* fruit, *Gardenia jasminoides* fruit
5	7.10	Luteolin	C_15_H_10_O_6_	[M − H] −	285.04	−1.0	*Forsythia suspensa* fruit
6	7.80	Baicalein	C_15_H_10_O_5_	[M − H] −	269.04	−1.5	*Pinellia ternata* root
7	8.20	Wogonin	C_16_H_12_O_5_	[M − H] −	283.06	−1.8	*Forsythia suspensa* fruit
8	9.73	Coniferin	C_16_H_22_O_8_	[M + H] +	341.12	2.2	*Pinellia ternata* root
9	10.42	Liquiritin	C_21_H_22_O_9_	[M + H] +	417.11	1.4	*Prunus armeniaca* seed
10	11.50	Licochalcone B	C_16_H_14_O_5_	[M − H] −	285.07	−1.6	*Prunus armeniaca* seed
11	14.33	Onjixanthone I	C_20_H_18_O_6_	[M + H] +	353.10	2.3	*Forsythia suspensa* fruit
12	15.88	2‐Monoolein	C_21_H_40_O_4_	[M + H] +	357.29	2.6	*Coix lacryma‐jobi* seed
13	17.10	β‐Amyrin acetate	C_32_H_52_O_2_	[M + H] +	469.40	2.7	*Forsythia suspensa* fruit
14	18.37	Hesperetin	C_16_H_14_O_6_	[M + H] +	301.07	0.9	Stephania tetrandra root
15	18.60	Ammidin	C_21_H_22_O_7_	[M + H] +	385.12	1.9	*Gardenia jasminoides* fruit
16	19.27	Glabridin	C_20_H_20_O_4_	[M + H] +	323.12	1.5	*Prunus armeniaca* seed
17	19.48	Cycloartenol	C_30_H_50_O	[M + H] +	427.39	2.8	*Coix lacryma‐jobi* seed
18	19.56	(‐)‐Phillygenin	C_21_H_24_O_6_	[M + H] +	371.15	1.3	*Forsythia suspensa* fruit
19	20.37	Mandenol	C_22_H_42_O_2_	[M + H] +	337.31	1.1	*Gardenia jasminoides* fruit, *Coix lacryma‐jobi* seed
20	21.08	Crocetin	C_20_H_24_O_4_	[M + H] +	327.16	1.2	*Gardenia jasminoides* fruit
21	21.12	Tetraneurin A	C_20_H_26_O_6_	[M − H] −	361.16	−2.5	Stephania tetrandra root
22	22.37	Linoleic acid	C_18_H_32_O_2_	[M − H] −	279.23	−0.9	*Gardenia jasminoides* fruit, *Coix lacryma‐jobi* seed, *Pinellia ternata* root
23	22.63	Beta‐sitosterol	C_29_H_50_O	[M + H] +	414.72	2.2	Stephania tetrandra root, *Forsythia suspensa* fruit, *Gardenia jasminoides* fruit, *Pinellia ternata* root
24	22.94	Oleic acid	C_18_H_34_O_2_	[M − H] −	281.24	−0.8	*Pinellia ternata* root
25	24.65	Stigmasterol	C_29_H_46_O	[M + H] +	413.37	2.2	*Prunus armeniaca* seed, *Gardenia jasminoides* fruit, *Coix lacryma‐jobi* seed, *Pinellia ternata* root
26	24.95	Geniposidic acid	C_16_H_22_O_10_	[M − H] −	373.11	−1.9	*Gardenia jasminoides* fruit

Abbreviations: m/z, mass‐to‐charge ratio; No., number; ppm, parts per million; RT, retention time.

### 3.4 Potential Targets and Pathways Associated With the Effects of XuanBi on CIA Synovial Pathological Changes

According to our previous study (Ma and Zhou 2024), a total of 154 differentially expressed proteins (DEPs) associated with CIA pathology were identified (three biological replicates, each with more than one unique peptide), exhibiting a fold change > 1.2 or < 0.83 (Figure 4A). Furthermore, using the TCMSP database, we retrieved all targets corresponding to the ingredients of XuanBi herbs and constructed an herb‐compound‐target interaction network (Figure 4B). Among these, 182 potential targets were matched to the 26 absorbable components detected in both XuanBi decoction and serum samples after oral administration. By intersecting these predicted targets with the synovial DEPs, we identified 14 common targets (Figure 4C, Table 2), suggesting that these proteins may be associated with the potential regulatory effects of XuanBi on CIA‐related synovial pathological changes. These 14 targets were subsequently imported into Cytoscape for Gene Ontology (GO) and Kyoto Encyclopedia of Genes and Genomes (KEGG) pathway enrichment analyses.

**FIGURE 4:**
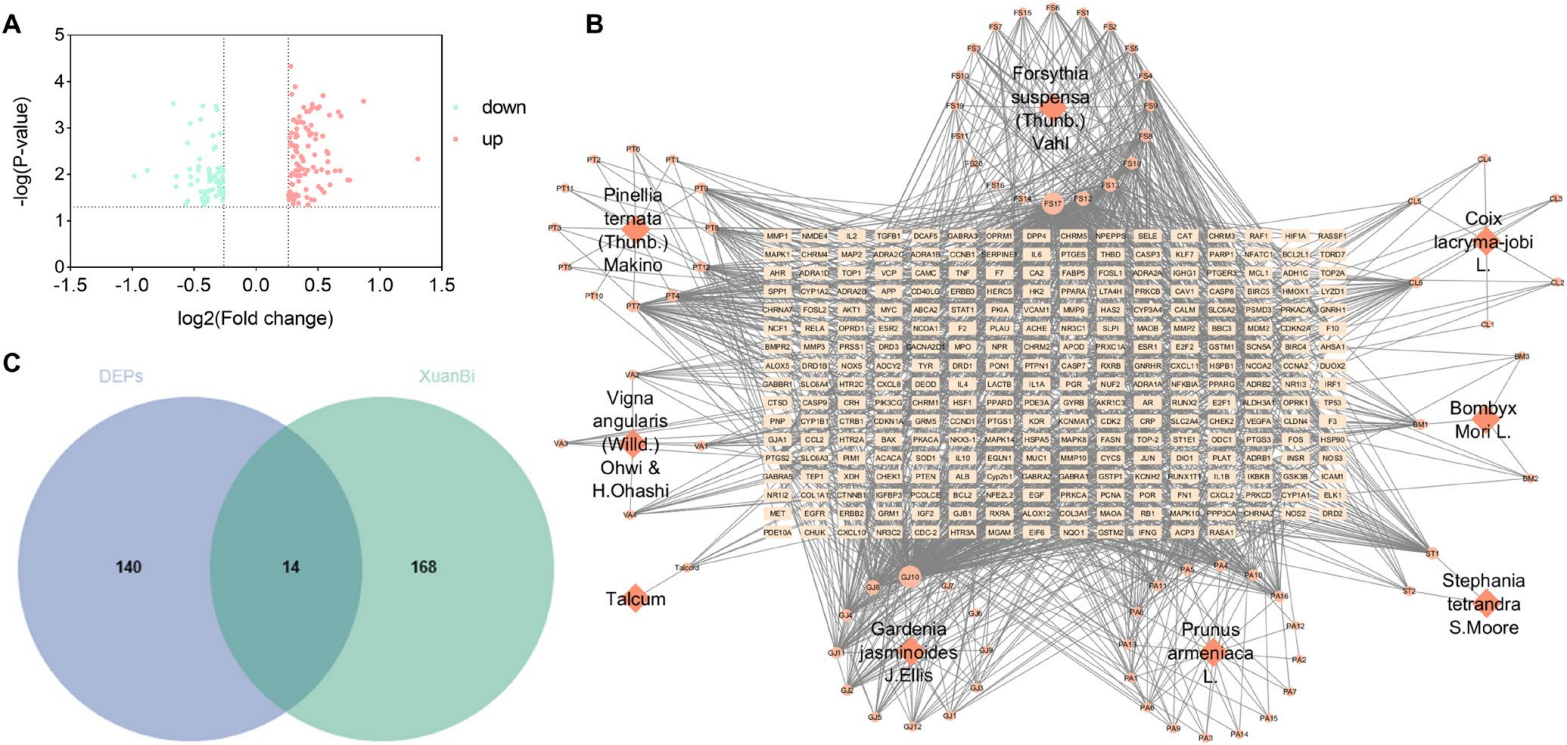
Prediction of potential target proteins associated with the effects of XuanBi herbal formula in arthritic synovium through integrated proteomics and network pharmacology analysis. (A) Comparison of differentially expressed proteins between the control and model groups, with upregulated proteins marked in red (log2 fold change > 0.263) and downregulated proteins in green (log2 fold change < −0.263), all meeting a significance threshold of *p* ≤ 0.05. (B) Herb (diamond)—Compound (circle)—Target (rectangle) network. (C) Intersection between XuanBi herbal formula's targets and differentially expressed proteins.

**TABLE 2:** The 14 common target proteins shared between the targets corresponding to the detected ingredients and the differentially expressed proteins (DEPs) in synovial tissue.

No.	Symbol	log10 (score)	Fold change	Ingredients	Source
1	FASN	3.58	0.62	(‐)‐Catechin	*Vigna angularis* seed
2	ACACA	2.01	0.64	Quercetin	*Forsythia suspensa* fruit, *Gardenia jasminoides* fruit
3	LTA4H	2.39	0.72	Stigmasterol	*Prunus armeniaca* seed, *Gardenia jasminoides* fruit, *Coix lacryma‐jobi* seed, *Pinellia ternata* root
4	AHSA1	2.14	0.76	Quercetin, wogonin	*Forsythia suspensa* fruit, *Gardenia jasminoides* fruit
5	PCOLCE	1.95	0.78	Quercetin	*Forsythia suspensa* fruit, *Gardenia jasminoides* fruit
6	SOD1	2.76	1.21	Quercetin, liquiritin	*Forsythia suspensa* fruit, *Gardenia jasminoides* fruit, *Prunus armeniaca* seed
7	PON1	2.13	1.22	Beta‐sitosterol, quercetin	Stephania tetrandra root, *Forsythia suspensa* fruit, *Gardenia jasminoides* fruit, *Pinellia ternata* root
8	PCNA	1.51	1.26	Luteolin	*Forsythia suspensa* fruit
9	MMP3	3.57	1.30	Quercetin	*Forsythia suspensa* fruit, *Gardenia jasminoides* fruit
10	CAV1	3.13	1.32	Quercetin	*Forsythia suspensa* fruit, *Gardenia jasminoides* fruit
11	MMP9	3.19	1.36	Baicalein, quercetin, luteolin	*Pinellia ternata* root, *Forsythia suspensa* fruit, *Gardenia jasminoides* fruit
12	FN1	3.83	1.45	Wogonin	*Forsythia suspensa* fruit
13	CA2	2.40	1.49	Coniferin, Licochalcone B	*Pinellia ternata* root, *Prunus armeniaca* seed
14	POR	1.91	1.51	Quercetin	*Forsythia suspensa* fruit, *Gardenia jasminoides* fruit

*Note:* The Score denotes the confidence metric for protein or peptide identification as determined by Mascot database searching; Fold change quantifies the expression ratio of synovial proteins in collagen‐induced arthritis (CIA) model rats versus normal control rats.

The GO analysis (Figure 5A) suggested that XuanBi may influence the proliferation of synoviocytes, inflammatory responses, and cell differentiation through the positive regulation of molecular functions. The identified candidate proteins were predominantly associated with extracellular matrix and cell membrane‐related components and with molecular functions including cell adhesion molecule binding, signaling receptor binding, and collagen binding. KEGG pathway analysis (Figure 5B) indicated that the 14 common targets were enriched in several pathways potentially associated with the effects of XuanBi on RA‐related synovial pathological changes, including ECM–receptor interaction, focal adhesion, and IL‐17 signaling pathways.

**FIGURE 5:**
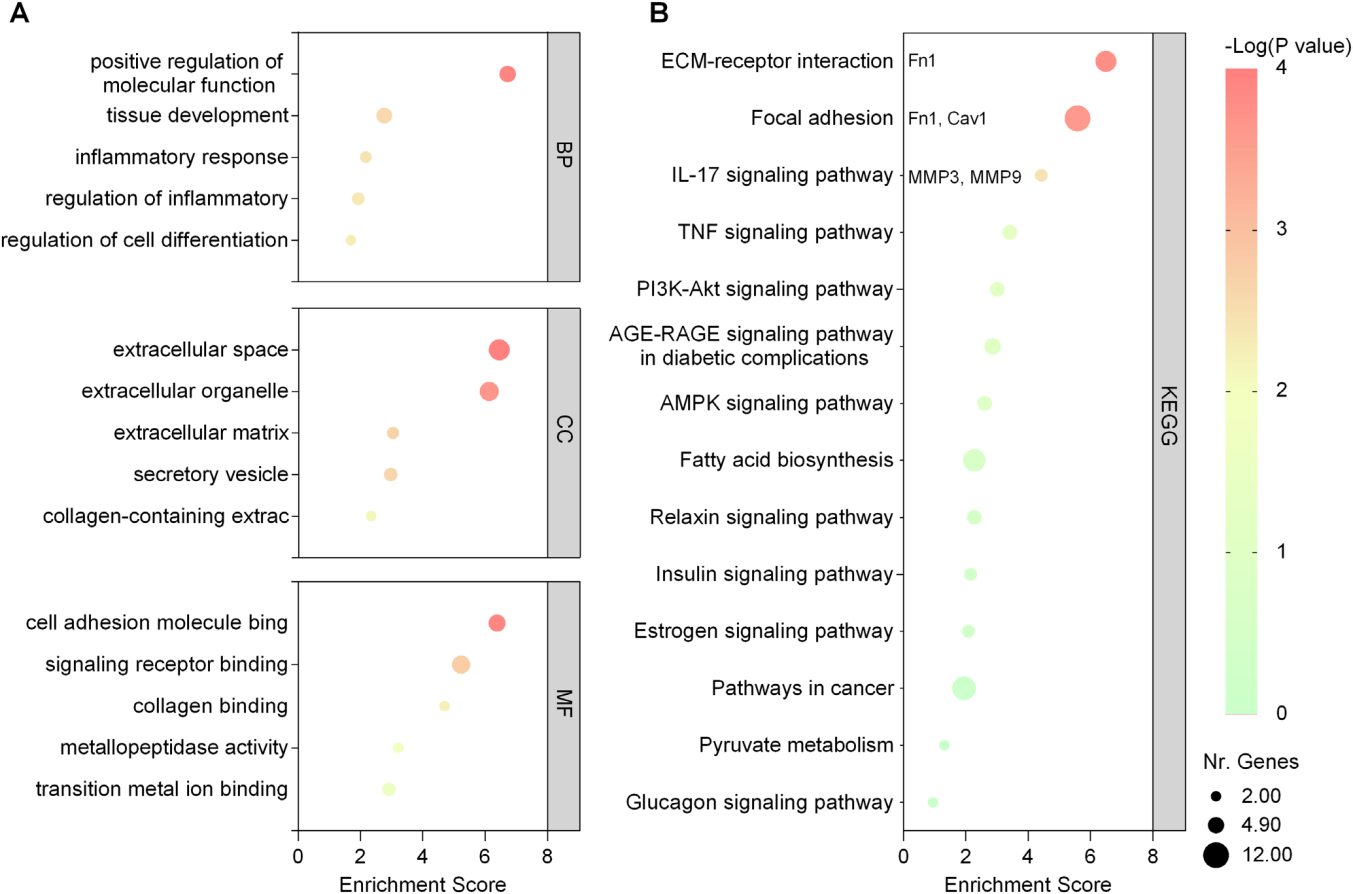
Bioinformatics analysis of potential target proteins. (A) Gene Ontology (GO) enrichment analysis of the 14 target proteins (BP, biological process; CC, cellular component; MF, molecular function). (B) Kyoto Encyclopedia of Genes and Genomes (KEGG) enrichment analysis of the 14 differentially expressed proteins (DEPs).

Based on the enrichment results and KEGG pathway annotations, we constructed a proposed schematic model to illustrate the potential association between XuanBi treatment and proteins involved in ECM‐receptor interaction, focal adhesion, and IL‐17 signaling (Figure 6). Within the proposed network, CAV1 was associated with FYN phosphorylation, which may be related to the proliferation and motility of RASFs. Additionally, FN1 was linked in the proposed network to the expression of ITGA2B and ITGB1 on the cell membrane and to the phosphorylation of tyrosine‐protein kinase SRC (p‐SRC) and focal adhesion kinase (p‐FAK). These changes may jointly affect the motility and proliferation of synoviocytes. Furthermore, the proposed IL‐17‐related network included Act 1 (p‐Act1), p38 mitogen‐activated protein kinase (p‐P38), and nuclear factor kappa B p65 subunit (p‐P65), together with MMP3, MMP9, and inflammatory factors including IL‐1β, IL‐6, and TNF‐α. These connections illustrate the potential relationships among proteins associated with synoviocyte activity and inflammatory signaling.

**FIGURE 6:**
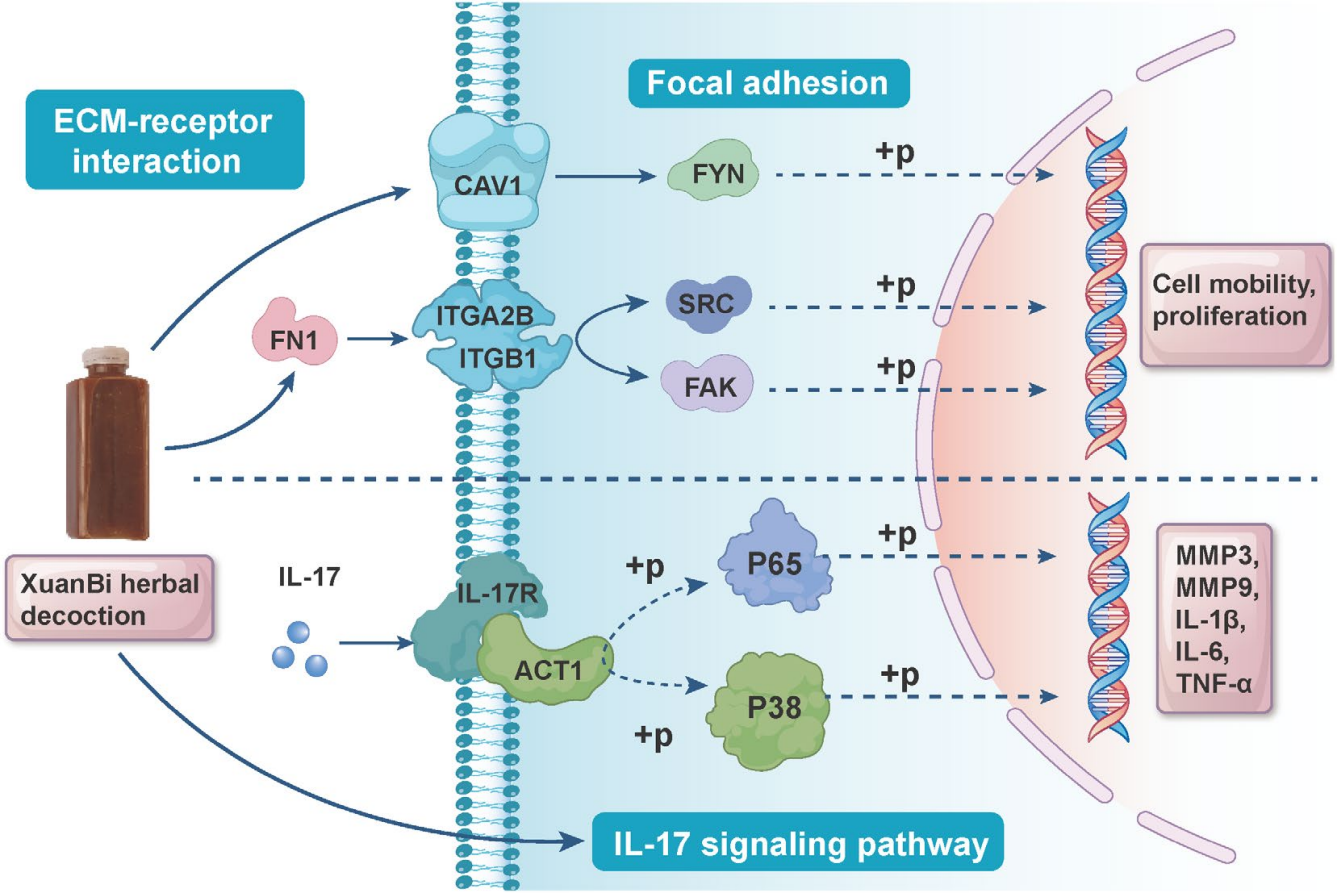
Proposed schematic model illustrating the potential association between XuanBi treatment and proteins involved in extracellular matrix (ECM)–receptor interaction, focal adhesion, and interleukin‐17 (IL‐17) signaling.

Taken together, these findings suggest that the effects of XuanBi on synovial inflammatory hyperplasia may be associated with changes in proteins involved in multiple signaling pathways.

### 3.5 XuanBi Inhibits the Expression of FN1, CAV1, MMP3, and MMP9 in CIA Synovium

On Day 28, FN1, CAV1, MMP3, and MMP9 were expressed to varying degrees in the synovium of each group (Figure 7). In the normal synovium, the expression levels of these proteins are minimal. In the arthritis model, which is characterized by pronounced synovial hyperplasia, the expression of these proteins was significantly increased. After XuanBi intervention, the expression levels of these proteins decreased with increasing doses. LD XuanBi had a negligible effect compared with the model group, MD XuanBi showed a more noticeable reduction, and HD XuanBi achieved the most substantial decrease. The use of MP as a positive control resulted in the most significant reduction in protein expression among all the treatments.

**FIGURE 7:**
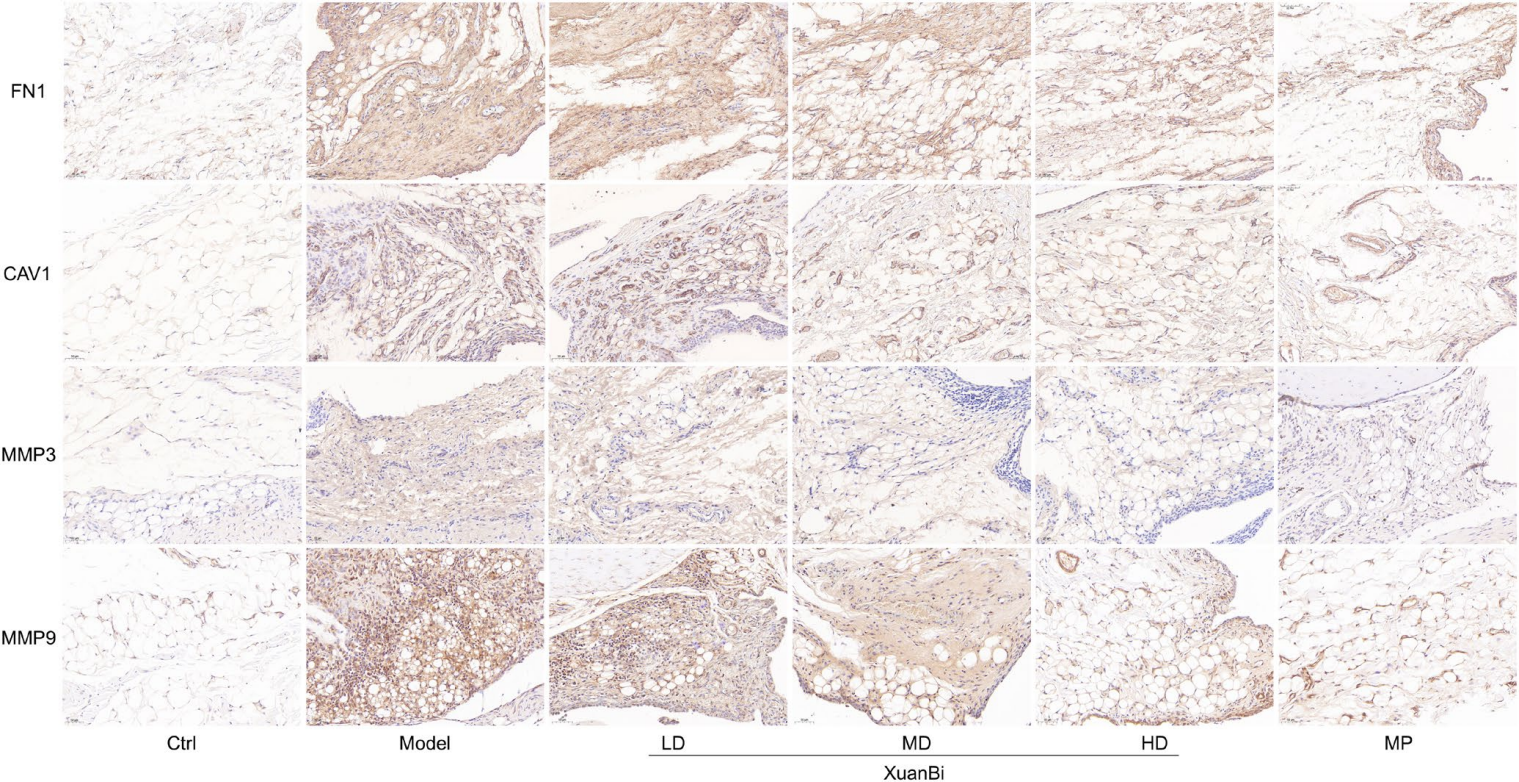
Representative expression of fibronectin 1 (FN1), caveolin 1 (CAV1), matrix metallopeptidase 3 (MMP3) and matrix metallopeptidase 9 (MMP9) in the synovial tissue of each group on Day 28: Control (Ctrl), arthritic model (Model), methylprednisolone intervention (MP), and XuanBi intervention at low dose (LD), medium dose (MD), or high dose (HD).

### 3.6 Identification Results of SFs and RASFs

Vimentin is a protein that is specifically expressed in fibroblast‐like synoviocytes. To confirm the identity of the isolated SF and RASF cells, we assessed Vimentin expression in synovial fibroblasts from normal and CIA model rats via immunofluorescence. In SFs and RASFs, the immunofluorescence results revealed that a significant majority of the cells exhibited green staining (Figure 8A), indicating positive vimentin expression. This confirms that the isolated cells are indeed SFs, with a purity exceeding 90%.

**FIGURE 8:**
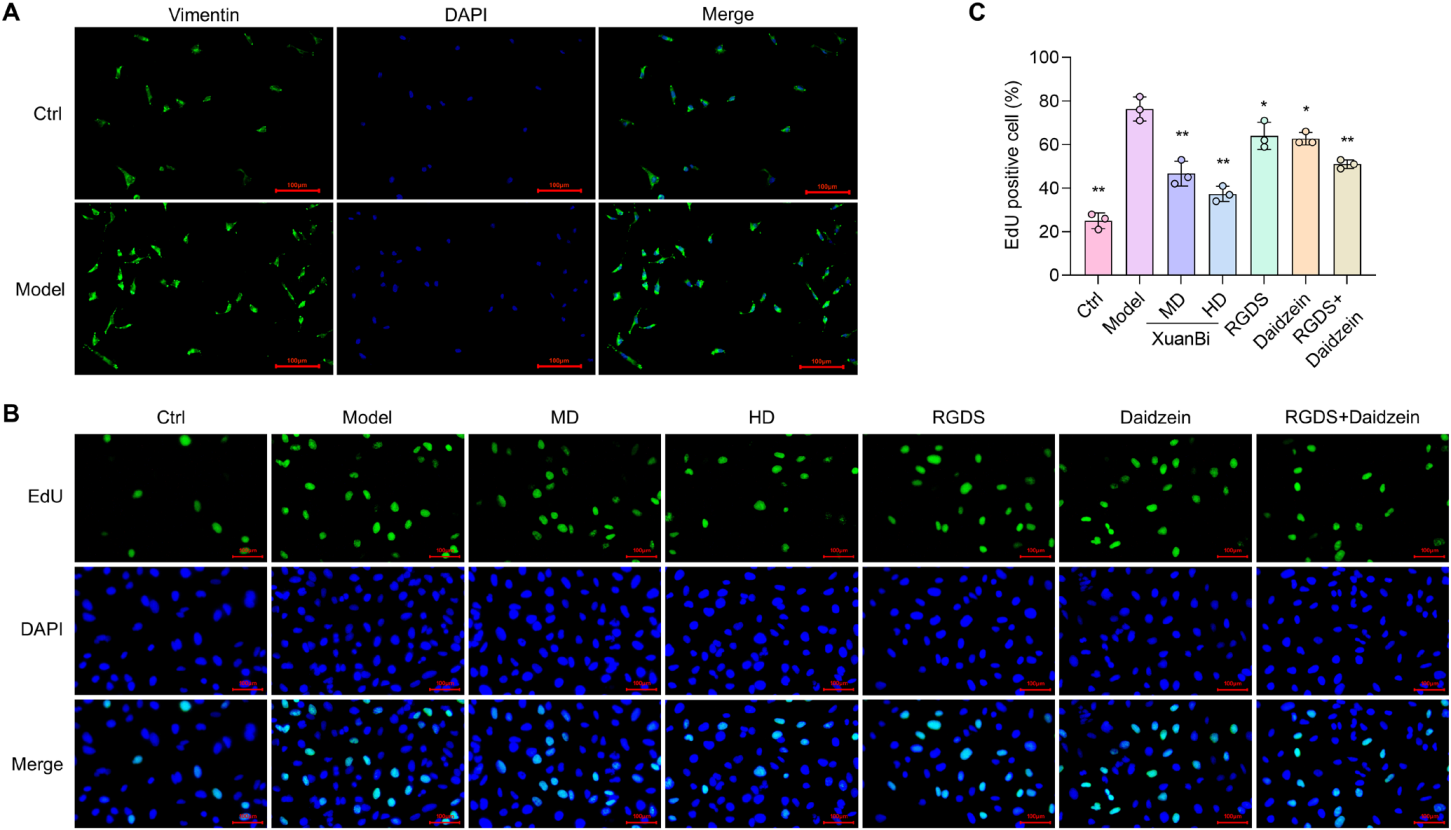
Identification of synovial fibroblasts and the effect of XuanBi herbal formula on their proliferation. (A) The expression of Vimentin in synovial fibroblasts (SFs) from control rats (Ctrl) and rheumatoid arthritis synovial fibroblasts (RASFs) from collagen‐induced arthritis rats (Model). (B, C) Effects of medium‐dose (MD) or high‐dose (HD) XuanBi, Arg‐Gly‐Asp‐Ser (RGDS, FN1 inhibitor), Daidzein (CAV1 inhibitor), or the combination of RGDS and Daidzein on the proliferation of RASFs. Statistical significance was determined by one‐way analysis of variance (ANOVA) followed by Dunnett's post hoc test for comparisons with the Model group. *p < 0.05; **p < 0.01.

Through animal experiments, we found that LD XuanBi was ineffective in suppressing arthritis symptoms and synovial inflammatory hyperplasia in CIA rats. Therefore, in the following cell experiments, we focused on the MD and HD of XuanBi to intervene in RASFs. Additionally, to further evaluate the potential involvement of FN1‐ and CAV1‐associated signaling, we used an FN1 inhibitor (fibronectin tetrapeptide (Yang et al. 2022), RGDS) at 75 μM and a CAV1 inhibitor (Daidzein; Nakashima et al. 2024) at 25 μM as positive controls to treat RASFs.

### 3.7 XuanBi Inhibits the Proliferation of RASFs

EdU staining and immunofluorescence results indicated that both MD and HD XuanBi interventions significantly reduced the proliferation rate of RASFs (Figure 8B,C), compared with that of the model group (*p* < 0.01). Treatment with RGDS or Daidzein led to a marked reduction in RASF proliferation compared with the model group (*p* < 0.05), albeit less effectively than XuanBi. Furthermore, the combined effect of RGDS and Daidzein was more significant (*p* < 0.01). Together with the immunohistochemical results showing that XuanBi reduced FN1 and CAV1 expression in synovial tissue, these findings suggest that the inhibitory effect of XuanBi on RASF proliferation may be associated with FN1/CAV1‐related pathway changes.

### 3.8 XuanBi Inhibits the Migration and Invasion of RASFs

MD and HD XuanBi treatments effectively inhibited RASF migration, invasion, and wound closure rates, showing a significant difference when compared to the model group (*p* < 0.01) (Figure 9A–D). In addition, the use of the RGDS or Daidzein resulted in reduced rates of cell migration, invasion, and healing for the RASFs (*p* < 0.05), although these were not as prominent as those induced by the use of XuanBi. It was also evident that the combination of the RGDS with Daidzein was highly potent (*p* < 0.01). Our data suggest that the reduced migration and invasion of RASFs after XuanBi treatment may be associated with modulation of FN1 and CAV1 expression.

**FIGURE 9:**
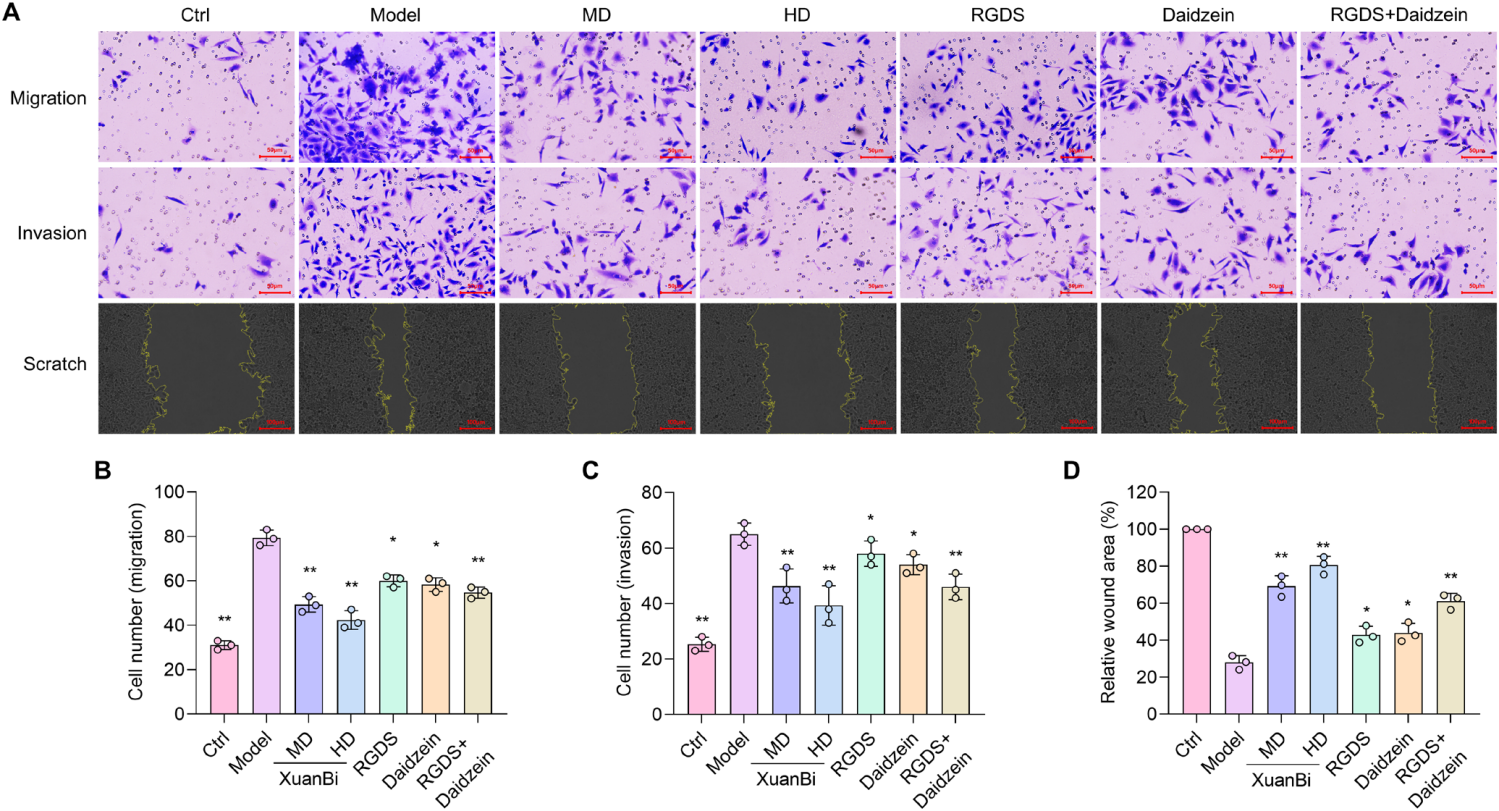
Transwell and wound healing assays were conducted to evaluate the migration and invasion of synovial fibroblasts (SFs) from control rats (Ctrl) and rheumatoid arthritis synovial fibroblasts (RASFs) from arthritic rats (Model), further assessing the effects of medium‐dose (MD) and high‐dose (HD) XuanBi, along with the FN1 inhibitor Arg‐Gly‐Asp‐Ser (RGDS) and the CAV1 inhibitor Daidzein, on the migration and invasion of RASFs (A). Quantitative analyses were performed for migration (B), invasion (C), and scratch assay (D). Statistical significance was determined by one‐way analysis of variance (ANOVA) followed by Dunnett's post hoc test for comparisons with the Model group. *p < 0.05; **p < 0.01.

### 3.9 XuanBi Inhibits ITGA2B/ITGB1 Expression in RASFs

From the analysis of the relevant pathways of XuanBi in the KEGG database, we found that FN1 is involved in the interaction between the ECM and receptors, which is related to the expression of integrins ITGA2B and ITGB1 on the surface of RASFs. To confirm the effect of XuanBi, we used FCM. Flow cytometry analysis showed that the model group had a notable increase in the proportion of ITGA2B+/ITGB1+ RASFs relative to the control, alongside a decrease in ITGA2B−/ITGB1− cells (Figure 10A). MD and HD XuanBi treatments effectively reduced the ITGA2B+/ITGB1+ population when compared to the model group (*p* < 0.01), producing an effect similar to that of RGDS (*p* < 0.01). These results indicate that the reduction in ITGA2B/ITGB1‐positive RASFs following XuanBi treatment was accompanied by changes in FN1 expression, consistent with potential involvement of the FN1‐associated ECM–receptor interaction pathway.

**FIGURE 10:**
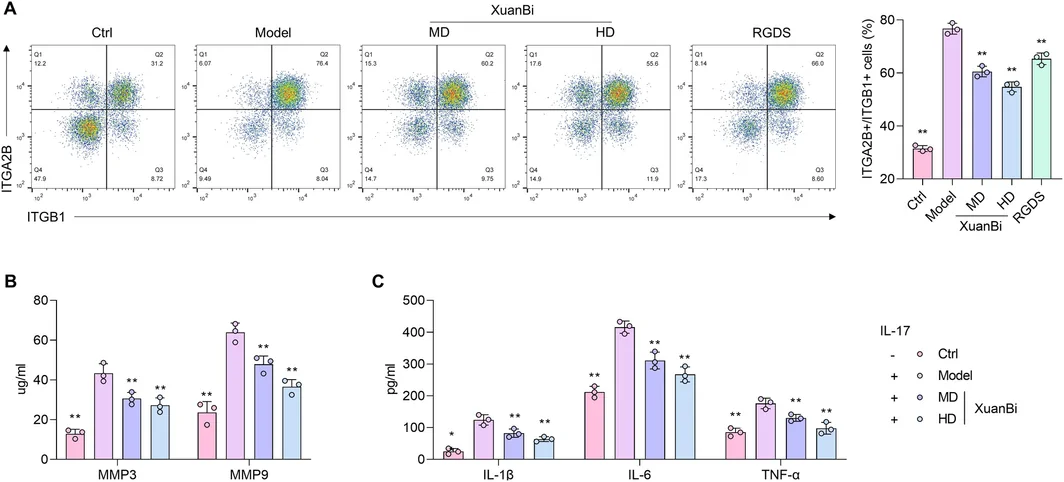
Flow cytometry was performed to evaluate ITGA2B/ITGB1 expression in synovial fibroblasts (SFs) from control rats (Ctrl) and rheumatoid arthritis synovial fibroblasts (RASFs) from arthritic rats (Model), and to assess the effects of medium‐dose (MD) or high‐dose (HD) XuanBi on integrin expression in RASFs (A). Additionally, enzyme‐linked immunosorbent assay (ELISA) was used to quantify matrix metallopeptidase 3 (MMP3), matrix metallopeptidase 9 (MMP9) (B), interleukin‐1 beta (IL‐1β), interleukin‐6 (IL‐6), and tumor necrosis factor alpha (TNF‐α) (C), in Ctrl and Model groups, further examining the impact of MD or HD XuanBi on these factors. Statistical significance was determined by one‐way analysis of variance (ANOVA) followed by Dunnett's post hoc test for comparisons with the Model group. *p < 0.05; **p < 0.01.

### 3.10 XuanBi Treatment Is Associated With Changes in ECM–Receptor Interaction, Focal Adhesion, and IL‐17 Signaling‐Related Proteins and Inflammatory Mediators in RASFs

We evaluated proteins associated with ECM–receptor interaction and focal adhesion signaling in each experimental group. Compared with the Model group, MD or HD XuanBi treatment significantly reduced FN1, p‐SRC, p‐FAK, CAV1, and p‐FYN levels (*p* < 0.05) (Figure 11A,B). Treatment with RGDS or Daidzein significantly reduced the activity of downstream targets of FN1 and CAV1, respectively, compared with the model group (*p* < 0.01). These findings indicate that XuanBi treatment was associated with reduced FN1 and CAV1 expression and decreased phosphorylation of proteins related to ECM–receptor interaction and focal adhesion signaling. Together with the observed reductions in RASF proliferation and migration, these molecular changes support the potential involvement of these pathways in the cellular effects of XuanBi.

**FIGURE 11:**
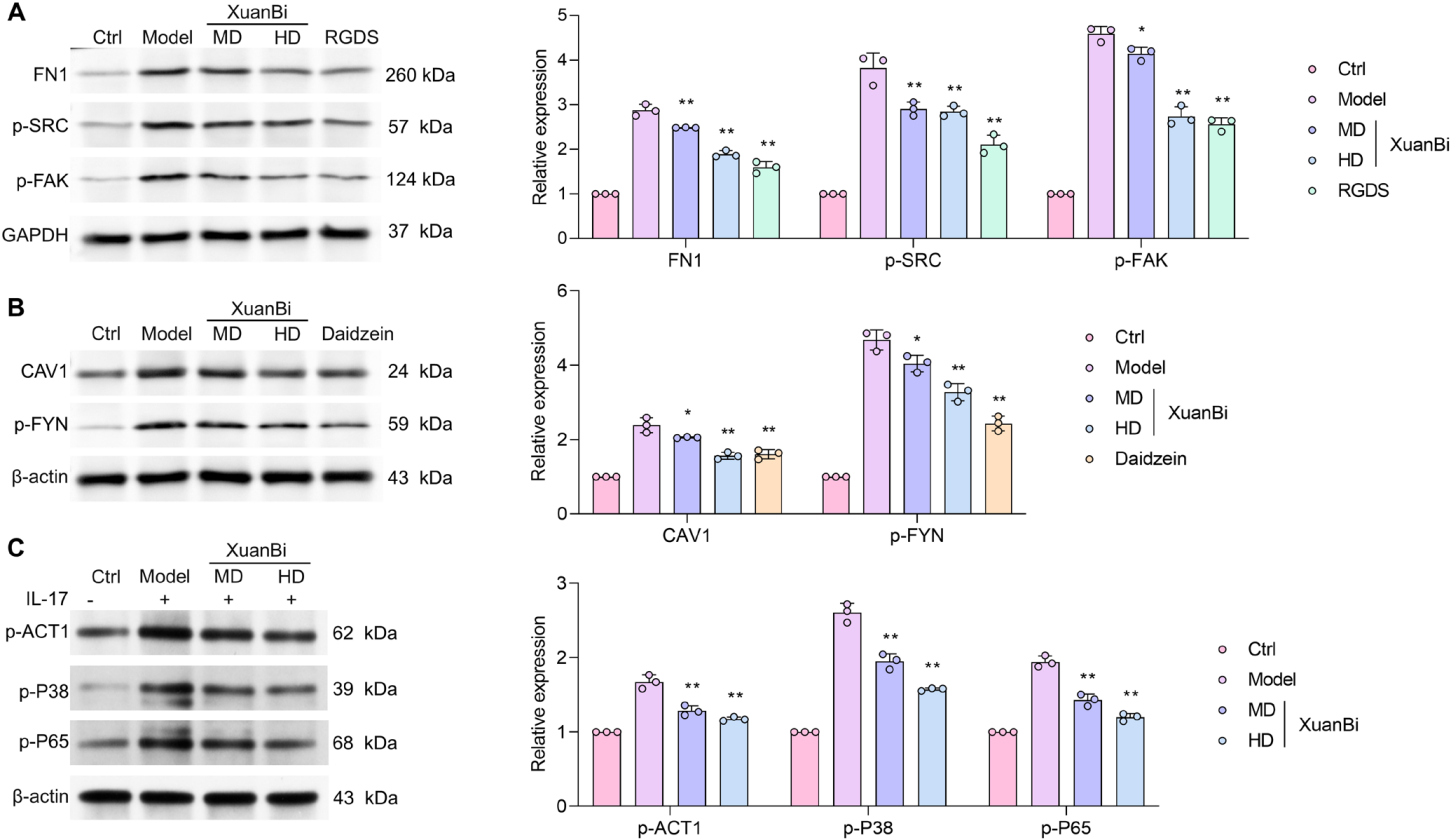
Western blotting was performed to evaluate proteins associated with extracellular matrix (ECM)–receptor interaction, focal adhesion, and interleukin‐17 (IL‐17) signaling in synovial fibroblasts (SFs) from control rats (Ctrl) and rheumatoid arthritis synovial fibroblasts (RASFs) from arthritic rats (Model), and to assess the effects of medium‐dose (MD) or high‐dose (HD) XuanBi treatment on these proteins in RASFs. (A) Quantitative analysis of fibronectin 1 (FN1), phosphorylated SRC (p‐SRC), and phosphorylated focal adhesion kinase (p‐FAK), which are associated with ECM–receptor interaction and focal adhesion signaling. (B) Quantitative analysis of caveolin 1 (CAV1) and phosphorylated FYN (p‐FYN). (C) Quantitative analysis of phosphorylated Act1 (p‐Act1), phosphorylated p38 mitogen‐activated protein kinase (p‐P38), and phosphorylated nuclear factor kappa B p65 (p‐P65) in RASFs following interleukin‐17A (IL‐17A) stimulation, with or without XuanBi treatment. Statistical significance was determined by one‐way analysis of variance (ANOVA) followed by Dunnett's post hoc test for comparisons with the Model group. *p < 0.05; **p < 0.01.

To evaluate the effect of XuanBi on the activated IL‐17 signaling pathway, RASFs were stimulated with IL‐17A (50 ng/mL) for 24 h to establish an inflammatory condition in vitro. Compared with the IL‐17A‐stimulated Model group, treatment with medium‐ or high‐concentration XuanBi significantly decreased the levels of p‐Act1, p‐P38, and p‐P65 (Figure 11C) (*p* < 0.05). Furthermore, the levels of IL‐17 pathway‐related inflammatory mediators, including MMP3, MMP9 (Figure 10B), IL‐1β, IL‐6, and TNF‐α (Figure 10C), were markedly reduced in the MD and HD groups compared with the IL‐17A‐stimulated Model group (*p* < 0.01). These findings indicate that both MD and HD XuanBi treatments suppressed IL‐17 pathway‐related inflammatory mediators in RASFs.

## 4 Discussion

This study demonstrated that MD and HD XuanBi formulations effectively alleviated ankle joint inflammation in CIA rats, reducing synovial hyperplasia and limiting inflammatory cell infiltration. These findings provide preclinical evidence supporting the potential anti‐arthritic effects of XuanBi in experimental RA (Hou et al. 2009). Given that synovial inflammatory proliferation is a key component of RA pathophysiology (Yan‐ming et al. 2023), the observed effects of XuanBi may be associated with attenuation of this pathological process. We employed network pharmacology and bioinformatics tools to identify candidate proteins and pathways potentially associated with the effects of XuanBi on RA‐related synovial pathology. Candidate proteins including FN1, CAV1, MMP3, and MMP9 were associated with these pathways and may be relevant to synoviocyte proliferation, migration, and inflammatory responses.

Compared with selecting candidate compounds solely on the basis of pharmacological databases, identifying constituents that actually enter the circulation after oral administration provides a more biologically relevant chemical basis for subsequent component–target analysis. In the present study, 26 absorbable constituents were identified, including several polyphenolic compounds such as catechin, quercetin, luteolin, baicalein, wogonin, hesperetin, and glabridin. Plant‐derived polyphenols possess broad anti‐inflammatory activities, and some of the compounds identified in this study have also demonstrated biological effects relevant to RA. For example, quercetin has been shown to inhibit IL‐17‐induced RANKL production in RA synovial fibroblasts, Th17 cell differentiation, and osteoclast formation, thereby attenuating RA‐associated bone destruction (Ghosh et al. 2024). Baicalein has been reported to suppress IL‐1β‐induced proliferation of human RA fibroblast‐like synoviocytes and reduce the activity of ERK, p38, and NF‐κB‐related signaling pathways (Anaparti et al. 2022). These findings provide pharmacological support for the possibility that the absorbable polyphenolic constituents of XuanBi may contribute to the regulation of synovial inflammation. However, entry into the circulation only indicates the possibility of systemic exposure. Qualitative detection in serum does not directly reflect absolute bioavailability, exposure level, half‐life, tissue distribution, or causal pharmacological contribution. These parameters require further investigation through quantitative pharmacokinetic and tissue distribution studies (Wang et al. 2019). In addition, the present study evaluated XuanBi as a complete formula and did not include polyphenol‐enriched fractions, polyphenol‐depleted preparations, or individual‐compound intervention groups. Therefore, the observed anti‐inflammatory and anti‐synovial hyperplasia effects cannot be specifically attributed to polyphenolic constituents. The contributions of non‐polyphenolic components and the possible additive or synergistic interactions among different constituents should also be considered. Future studies should therefore distinguish the major pharmacologically active constituents of XuanBi through systematic fractionation and component‐level validation, with particular attention to the absorbable polyphenolic compounds identified in the present study. Evaluation of individual polyphenols, polyphenol‐enriched fractions, and defined combinations of these constituents may help determine their independent, additive, or synergistic contributions to the observed anti‐inflammatory and anti‐synovial hyperplasia effects.

In the case of the ECM‐receptor interaction and Focal adhesion pathways, FN1 and CAV1 have key functions in modulating cell‐ECM adhesion and intracellular signaling, respectively (Hwang et al. 2004; Sun et al. 2024). FN1, which is present in the extracellular matrix, has been reported to interact with integrins such as ITGA2B and ITGB1 to trigger a series of events that activate downstream proteins such as SRC and FAK (Schlaepfer et al. 2023; Ma et al. 2020). This activation, mediated by phosphorylation, is crucial for relaying ECM information into the cell and associating it with the cytoskeleton. These processes are basic to the function of synoviocytes, especially in relation to proliferation and motility (Guo et al. 2023), which could contribute to synovial hyperplasia in RA. On the other hand, CAV1, a membrane‐associated protein, is integral to the structure of caveolae, which are small invaginations that support various cellular processes (Ren et al. 2023). CAV1 can influence cell barrier function by facilitating the phosphorylation of FYN (Hui‐min et al. 2020), which in turn may impact cell proliferation and mobility (Chen et al. 2023). In this study, both medium‐dose and high‐dose XuanBi significantly reduced the proliferative ability, migration, and invasion potential of RASFs. These effects were accompanied by decreased FN1 and CAV1 expression and altered phosphorylation of SRC, FAK, and FYN. These parallel changes are consistent with potential involvement of FN1/CAV1‐associated ECM–receptor interaction and focal adhesion signaling, although direct causal relationships remain to be established. RGDS or Daidzein alone showed weaker inhibitory effects on RASF proliferation and motility than XuanBi treatment. Combined RGDS and Daidzein treatment showed stronger inhibitory effects than either inhibitor alone.

The IL‐17 signaling pathway is a well‐known mediator of inflammation in RA (Cui et al. 2025), with key molecules such as Act1, P38, and P65 being involved in the inflammatory response (Kim et al. 2019). Upon IL‐17 receptor activation, Act1 participates in downstream signaling that involves kinases such as p38 (Ru et al. 2014). Activation of p38 signaling has been associated with increased production of inflammatory mediators, including the matrix metalloproteinases MMP3 and MMP9, which may contribute to persistent synovial inflammation (Zhu et al. 2022). Similarly, p65, a component of the NF‐κB complex, participates in inflammatory signaling and, following activation and nuclear translocation (Gu et al. 2024), contributes to the transcriptional regulation of inflammatory cytokines (Zhu et al. 2024), including IL‐1β, IL‐6, and TNF‐α. These inflammatory mediators are closely associated with synovial inflammation and may participate in downstream inflammatory responses related to IL‐17 signaling (Zafari et al. 2019; Sharma et al. 2012). In the present study, XuanBi treatment significantly reduced the phosphorylation levels of Act1, p38, and p65 in IL‐17A‐stimulated RASFs. These changes were accompanied by reduced levels of inflammatory mediators, supporting a potential association between XuanBi treatment and attenuation of IL‐17‐related inflammatory signaling. MMP3 and MMP9 are involved in extracellular matrix and cartilage degradation in RA (Shah et al. 2024), and MMP3 has also been associated with disease activity and structural damage (Müller‐Ladner et al. 2007). Therefore, the reduced levels of MMP3 and MMP9 observed after XuanBi treatment may be relevant to the attenuation of joint pathological changes in the experimental model. We also observed reduced concentrations of IL‐1β, IL‐6, and TNF‐α following XuanBi treatment, consistent with its anti‐inflammatory effects in the experimental model. TNF‐α is an important pro‐inflammatory mediator involved in inflammatory responses in RA (Yue et al. 2020). IL‐1β also plays an important role in sustaining chronic inflammatory responses (Armaka et al. 2022). Their reduction following XuanBi treatment is therefore consistent with an attenuation of the inflammatory phenotype observed in the experimental model. Collectively, these findings suggest that IL‐17‐related signaling may be involved in the anti‐inflammatory effects associated with XuanBi treatment in the experimental model. However, because direct IL‐17 pathway blockade or rescue experiments were not performed, these findings support the potential involvement of IL‐17‐related signaling rather than establishing it as a direct causal mechanism of XuanBi action.

Integrating network pharmacology and bioinformatics with proteomics has proven highly effective for elucidating the mechanisms of traditional herbal medicines (Wu et al. 2021), with numerous applications in the study of Chinese herbal formulas (Jia‐kang et al. 2022; Pang et al. 2023). These findings are further supported by the results obtained in the present study on the XuanBi herbal formula. This approach facilitates the identification of key proteins through integrated proteomic and pharmacological analyses (Zhang et al. 2024). Our results suggest that the effects of XuanBi may be associated with modulation of ECM–receptor interaction, focal adhesion, and IL‐17 signaling‐related proteins. Notably, FN1, CAV1, MMP3, and MMP9 were identified as candidate proteins associated with pathways such as ECM–receptor interaction, focal adhesion, and IL‐17 signaling, and these proteins may be involved in the regulation of synovial inflammatory hyperplasia in RA. By integrating these analyses with proteomics, we identified several pathways and candidate proteins associated with the effects of XuanBi on RASF proliferation, migration, and inflammatory responses, providing a framework for further mechanistic investigation.

It should also be noted that this study has several limitations. First, the CIA rat model cannot fully reproduce the complexity and heterogeneity of human RA synovial pathology, and validation using human RA synovial tissues or patient‐derived primary cells is still needed. In addition, although both male and female rats were included and sex distribution was balanced across groups as far as possible, the study was not designed to evaluate sex‐specific effects. Because biological sex may influence immune and inflammatory responses in experimental arthritis, the use of mixed‐sex animals without sex‐stratified analysis represents an additional limitation. Second, the experimental validation mainly focused on RASFs, whereas the involvement of immune cells and fibroblast‐immune cell interactions in the synovial microenvironment was not fully investigated. Third, because XuanBi is a multi‐herb formula with complex chemical composition, the present study did not distinguish the major pharmacologically active components from other absorbable constituents, nor did it determine whether these components act independently or synergistically. Although several absorbable polyphenolic compounds with reported anti‐inflammatory activities were identified, the present data do not establish that these polyphenols are the principal active constituents responsible for the observed effects. Future studies should evaluate individual absorbable components, with particular emphasis on the identified polyphenols, as well as polyphenol‐enriched fractions and defined component combinations, to determine their relative contributions and potential synergistic interactions. Such studies may also help clarify whether dietary or plant‐derived polyphenols contribute substantially to the pharmacological activity of the complete XuanBi formula. In addition, TCMSP‐based target prediction may be affected by database coverage and prediction bias. Fourth, the current data support pathway association and functional relevance but do not fully establish direct causal mechanisms. Further studies involving IL‐17A blockade, IL‐17 receptor‐level validation, and FN1/CAV1 genetic manipulation or rescue experiments are required to establish direct causal mechanisms. Finally, human clinical sample validation is needed before the findings can be translated into RA management.

## Author Contributions

**Hao Zhu:** conceptualization, methodology, investigation, writing – original draft. **Zhenfeng Pan:** conceptualization, methodology, investigation, writing – original draft. **Ying Lu:** validation, software, formal analysis, data curation. **Ping Xia:** software, data curation, validation, investigation. **Weiming He:** data curation, visualization, formal analysis, software. **Jingqi Zeng:** visualization, validation, software, data curation. **Fan Wang:** resources, supervision, project administration, funding acquisition, writing – review and editing, conceptualization. **Xiao Liu:** funding acquisition, writing – review and editing, project administration, resources, supervision, conceptualization. **Minggang Wei:** conceptualization, funding acquisition, writing – review and editing, project administration, resources, supervision.

## Funding

Funding support for this study was obtained from Suzhou Applied Basic Research (Medical and Health) and Technology Innovation Project (SYWD2024278, SYWD2024317), Research Projects of Jiangsu Provincial Association of Chinese Medicine (PDJH2024040, ZXFZ2024073, CYTF2024042), Jiangsu Province Leading Talents Cultivation Project for Traditional Chinese Medicine (SLJ0330); Youth project of TCM science and technology development plan of Jiangsu Province (QN202525); 2024 Suzhou Science and Education Driven Healthcare Enhancement Project (MSXM2024006); Key project of TCM science and technology development plan of Suzhou Municipal (ZYZD202601); Hunan Provincial Natural Science Foundation (2023JJ30469); National Natural Science Foundation of China (82004290); Jiangsu Provincial Medical Innovation Center (CXZX202233); The First Batch of Outstanding Medical Leading‐Talent Teams of Jiangsu Province.

## Conflicts of Interest

The authors declare no conflicts of interest.

## Data Availability

The data that support the findings of this study are available on request from the corresponding author. The data are not publicly available due to privacy or ethical restrictions.
